# The number of procedures required to eliminate positioning nystagmus in benign paroxysmal positional vertigo

**DOI:** 10.1016/S1808-8694(15)31247-7

**Published:** 2015-10-20

**Authors:** Schaffeln Dorigueto Ricardo, Malavasi Ganança Maurício, Freitas Ganança Fernando

**Affiliations:** Master in Sciences, Program of Post-graduation in Otorhinolaryngology and Head and Neck Surgery, Federal University of Sao Paulo – Escola Paulista de Medicina, Otorhinolaryngology; Full Professor, Discipline of Otoneurology, UNIFESP-EPM, Full Professor, Discipline of Otorhinolaryngology, UNIFESP-EPM; Ph.D. in Medicine, Program of Post-graduation in Otorhinolaryngology and Head and Neck Surgery, Federal University of Sao Paulo – Escola Paulista de Medicina

**Keywords:** vertigo, vestibular diseases, rehabilitation, nistagmus

## Abstract

**Aim:**

To evaluate the number of weekly canalith repositioning procedures needed to eliminate positioning nystagmus in patients with benign paroxysmal positional vertigo and to verify influences of canalithiasis or cupulolithiasis and/or semicircular canal involvement.

**Study design:**

clinical prospective with transversal cohort.

**Material and Method:**

Sixty patients with benign paroxysmal positional vertigo were consecutively selected according to each combination of canalithiasis or cupulolithiasis with semicircular canal involvement. Patients were treated by means of canalith repositioning procedures repeated weekly until the elimination of the positioning nystagmus. Analysis of Variance was used to verify differences between the variables.

**Results:**

An average of 2.13 procedures (from 1 to 8) was needed to eliminate the positioning nystagmus. Canalithiasis required an average of 1.53 procedures, while cupulolithiasis needed 2.92 procedures (p = 0.0002). An average of two procedures was needed to eliminate the positioning nystagmus in cases with posterior canal involvement, 2.39 procedures in cases with anterior canal involvement and 2.07 procedures in cases with lateral canal involvement (p = 0.5213).

**Conclusions:**

From one to eight weekly canalith repositioning procedures were needed, with an average of two, to eliminate positioning nystagmus in benign paroxysmal positional vertigo. Cupulolithiasis requires a greater number of procedures than canalithiasis to eliminate positioning nystagmus. Semicircular canal involvement didn't influence the number of therapeutic maneuvers.

## INTRODUCTION

A benign paroxysmal positional vertigo (BPPV) is a biomechanical disorder of the vestibular labyrinth in which one or more semicircular canals are inappropriately stimulated at some head movements and/or positions, resulting in brief episodes of positional vertigo^1^. The characteristic complaint is marked rotation dizziness when the head position changes, when lying down, standing up or looking upwards by tilting the head backwards, which may be followed by nausea, vomiting and positioning nystagmus^2^.

Despite the high prevalence, BPPV is little diagnosed. It is the most common cause of vertigo owing to peripheral vestibular dysfunction, representing about 19% of the causes of vertigo^2^. Froehling et al.^3^ estimated the prevalence of 107 cases for 100,000 inhabitants/year.

This dysfunction occurs as an idiopathic form in many patients, but it may be secondary to inner ear traumatism, labyrinthitis, vestibular neuritis, circulatory failure in distribution of the anterior vestibular artery, use of ototoxic drugs and endolymphatic hydropsis, among other causes^2^.

Vertigo and the other clinical characteristics of BPPV are caused by debris of statoconia from the utricular macula, which may move abnormally and excite the receptors of semicircular ducts. There are two theories about the likely pathophysiological substrate of BPPV: ductolithiasis, which corresponds to the accumulation of statoconia fractions resulting from the utriculus and that are free in the endolymphatic circulation of one of the semicircular ducts, and cupulolithiasis, related to the accumulation of statoconia fractions from the utriculus adhered to the cupula of the ampullar crest of one of the semicircular ducts^4^^,^^5^.

The investigation of positioning nystagmus by observing direction and duration of ocular movement allows the identification of the affected canal, damaged labyrinth and distinction between cupulolithiasis or ductolithiasis, guiding treatment. The differentiation between ductolithiasis and cupulolithiasis is made by duration of positioning nystagmus for vertical semicircular canal (anterior and posterior) and direction of nystagmus for lateral semicircular canal^6^.

In past years, the application of specific repositioning maneuvers for statoconia for management of BPPV has given rise to special interest owing to its easy applicability and good results. Such maneuvers aim at removing the debris from statoconia, placed in the ducts of cupula of the semicircular canals towards the vestibule, following the ampuliphugal movement. Given that floating debris in the endolymph have higher density than circulating endolymph, they may be removed by a non-invasive way using a sequence of head movements towards gravity^6^.

Treatment strategies, based on statoconia repositioning, should be specific for the affected semicircular canal and the pathophysiological substrate^7^^,^^8^.

A controversial variable is the number of statoconia repositioning maneuvers necessary to convert positive Dix, Hallpike test into a negative one concerning the presence of positioning nystagmus. According to Vrabec^9^, the number of necessary maneuvers to induce remission of BPPV is variable and multiple treatments may be necessary in more than one third of the total patients.

The purpose of the present study was to assess the number of weekly maneuvers of statoconia repositioning required to eliminate the positioning nystagmus in patients with BPPV and to check the possible influences of the pathophysiological substrate and/or the semicircular canal affected in the number of therapeutic maneuvers.

## MATERIAL AND METHOD

The research study was analyzed and approved by the Research Ethics Committee, Hospital Sao Paulo – Escola Paulista de Medicina (UNIFESP). It was a prospective study which assessed 60 patients with diagnostic hypothesis of BPPV in the age range 17 to 83 years, Caucasian, including 35 female patients and 25 male patients.

We included patients with BPPV that presented vertigo and positioning nystagmus characteristic of the disease (latency, limited duration and fatigue at repetition of the maneuver that caused the nystagmus) at Dix, Hallpike test. Exclusion criteria were: 1) signs or symptoms of central impairment; 2) auditory impairment, unless considered compatible with presbycusis; 3) simultaneous impairment of more than one semicircular canal; 4) affection to the cervical spine or neck that prevented performance of diagnostic or therapeutic measurement; 5) patients that presented only vertigo when performing the diagnostic maneuver; 6) use of drugs that could have influenced the vestibular system.

Patients were submitted to otoneurological assessment that included anamnesis, ENT examination, pure tone audiometry, vocal discrimination, immittanciometry, static and dynamic balance investigation, and computed electronystagmography (ENG).

To investigate the positioning nystagmus, we performed Dix, Hallpike test. The test was started by the triggering position of vertigo and/or nystagmus, according to the information collected from the patient. If the patient did not know what position was responsible for triggering vertigo, the maneuver was started from the right side.

Nystagmus responses in each patient were observed using Frenzel lenses. The sixty patients were classified according to pathophysiological substrate and the involved semicircular canal, indicated by the triggering position of the nystagmus, duration and direction^6^.

Patients were selected in the study complying with a consecutive order of inclusion for each association between affected semicircular canal and pathophysiological substrate suspected in the diagnostic test. Thus, we formed consecutive groups for: 1. ductolithiasis of anterior canal; 2. cupulolithiasis of anterior canal; 3. ductolithiasis of lateral canal; 4. cupulolithiasis of lateral canal; 5. ductolithiasis of posterior canal, and 6. cupulolithiasis of posterior canal.

All patients were treated using statoconia repositioning maneuvers according to the pathophysiological substrate and affected semicircular canal. For the posterior canal, we used modified Epley maneuver, without the use of bone vibrator in the mastoid and without sedation of the patient, as described by Parnes, Prince-Jones^10^. For the lateral canal, we used Lempert^11^ maneuver. For the anterior canal, we used Epley maneuver modified for the anterior canal, as described by Herdman, Tusa^1^.

The first maneuver was performed immediately after the otoneurological assessment. The patient was instructed to come back every week, and a weekly maneuver was performed up to completely disappearance of positioning nystagmus.

The number of statoconia positioning maneuvers required to cause disappearance of positioning nystagmus was quantified.

The statistical analysis was performed using Variance Analysis (ANOVA) to check the differences between variables that comprised the affected semicircular canal (anterior, lateral or posterior canal) and pathophysiological substrate (ductolithiasis and cupulolithiasis) and the combination of these factors relative to the number of statoconia repositioning maneuvers required to make the positioning nystagmus disappear at Dix, Hallpike test in patients with benign paroxysmal positional vertigo. Results that had 5% significance were marked with an asterisk (*).

## RESULTS

The distribution of patients according to the pathophysiological substrate and affected semicircular canal is demonstrated in Table 1. We observed that the hypothesis of ductolithiasis was formulated in 34 patients (56.7%) and cupulolithiasis in 26 (43.3%).

**Table 1:** Distribution of 60 patients with benign paroxysmal positional vertigo according to pathophysiological substrate and affected semicircular canal.

Pathophysiologic al Substrate	Posterior Canal		Anterior Canal		Lateral Canal		Total	
	N	%	N	%	N	%	N	%
Ductolithiasis	16	57,1	10	55,6	8	57,1	34	56,7
Cupulolithiasis	12	42,9	8	44,4	6	42,9	26	43,3
Total	28	100,0	18	100,0	14	100,0	60	100,0

When ductolithiasis was the pathophysiological hypothesis, we found affection of right posterior semicircular canal in 8 patients (23.5%), left posterior semicircular canal in 8 patients (23.5%), right anterior semicircular canal in 6 patients (17.6%), left anterior semicircular canal in 4 patients (11.8%), right lateral semicircular canal in 4 patients (11.8%), and left lateral semicircular canal in 4 patients (11.8%).

When cupulolithiasis was the pathophysiological hypothesis, we observed affection of right posterior semicircular canal in 6 patients (23.1%), left posterior semicircular canal in 6 patients (23.1%), right anterior semicircular canal in 4 patients (15.4%), left anterior semicircular canal in 4 patients (15.4%), right lateral semicircular canal in 3 cases (11.5%) and left lateral semicircular canal in 3 patients (11.5%).

The number of weekly statoconia positioning maneuvers required to make the positioning nystagmus disappear in the 60 patients with BPPV at Dix, Hallpike test according to pathophysiological substrate and affected semicircular canal is described in Chart 1.

**Chart 1:** Number of weekly statoconia repositioning maneuvers required to eliminate positioning nystagmus at Dix, Hallpike test (1952) in 60 cases of Benign Paroxysmal positional vertigo according to pathophysiological substrate and affected semicircular canal.

Pathophysiological substrate and affected semicircular canal.	Number of MREs required to eliminate positioning nystagmus
Ductolithiasis CPD	One out of seven cases
(N = 8)	Two out of one case
Ductolithiasis CPE	One out of six cases
(N = 8)	Two out of two cases
Ductolithiasis CAD	One out of three cases
(N = 6)	Two out of three cases
Ductolithiasis CAE	One out of one case
(N = 4)	Two out of two cases
	Three out of one case
Ductolithiasis CLD	One out of two cases
(N = 4)	Two out of two cases
Ductolithiasis CLE	One out of one case
(N = 4)	Three out of three cases
Cupulolithiasis CPD	One out of two cases
(N = 6)	Three out of three cases
	Eight out of one case
Cupulolithiasis CPE	Two out of three cases
(N = 6)	Four out of three cases
Cupulolithiasis CAD	Three out of two cases
(N = 4)	Four out of two cases
Cupulolithiasis CAE	Two out of two cases
(N = 4)	Three out of one case
	Five out of one case
Cupulolithiasis CLD	Two out of two cases
(N = 3)	Three out of one case
Cupulolithiasis CLE	One out of two cases
(N = 3)	Four out of one case

**Key:** MREs: statoconia repositioning maneuvers; CPD: Right posterior semicircular canal; CPE: Left posterior semicircular canal; CAD: Right anterior semicircular canal; CAE: Left anterior semicircular canal; CLD: Right lateral semicircular canal; CLE: Left lateral semicircular canal.

When we compared the number of statoconia repositioning maneuvers required to make the positioning nystagmus disappear, without distinction of affected semicircular canal and pathophysiological substrate, we detected that 24 patients (40.0%) required one maneuver, 17 patients (28.3%) required two maneuvers, 11 patients (18.3%) required three maneuvers, six patients (10%) required four, one patient (1.7%) required five, and one patient (1.7%) required eight maneuvers. The mean number of maneuvers required to make positioning nystagmus disappear was 2.13 per patient.

Table 2 evidences mean and standard deviation of number of statoconia positioning maneuvers required to make the positioning nystagmus disappear, according to pathophysiological substrate and affected semicircular canal. In posterior and anterior semicircular canals, ductolithiasis required an average of one to two maneuvers and cupulolithiasis required on average 3 maneuvers. In the lateral semicircular canal, both ductolithiasis and cupulolithiasis required on average two maneuvers to make positioning nystagmus disappear.

**Table 2:** Means and standard deviations of number of repositioning maneuvers required to cause remission of positioning nystagmus at Dix, Hallpike test according to pathophysiological substrate and affected semicircular canal.

Posterior Canal		Anterior Canal	Lateral Canal
	Mean 1,19	1,70	2,00
Ductolithiasis	Dp 0,40	0,67	0,93
	N 16,00	10,00	8,00
	Mean 3,08	3,25	2,17
Cupulolithiasis	Dp 1,88	1,04	1,17
	N 12,00	8,00	6,00

**Key:** SD: standard deviation; n: number of patients.

All in all, on average, 1.53 maneuvers were required to make positioning nystagmus disappear in cases of ductolithiasis and 2.92 maneuvers in cases of cupulolithiasis.

In sum, on average two maneuvers were required for cases that affected the posterior canal, 2.39 maneuvers for cases of anterior canal affection, and 2.07 maneuvers in cases of lateral canal affection.

Considering the factor for ANOVA pathophysiological substrate, we detected the need for smaller number of repositioning maneuvers increases of ductolithiasis compared to cases of cupulolithiasis to make positioning nystagmus disappear at Dix, Hallpike test, reaching statistically significant difference (p = 0.0002*), as described in Table 3.

**Table 3:** Influence of variables that comprise the pathophysiological substrate (ductolithiasis and cupulolithiasis) and semicircular canal (anterior canal, lateral canal and posterior canal) and the combination of these factors and number of statoconia repositioning maneuvers required to eliminate the positioning nystagmus in patients with BPPV.

Factors and Association of Factors	F	Significance of F (p)
Factor pathophysiological substrate (Ductolithiasis / Cupulolithiasis)	16,169	0,0002^*^
Factor semicircular canal (CA /CL /CP)	0,659	0,5213
Factor pathophysiological substrate x Factor semicircular canal	2,873	0,0652

**Key:** TABLE ANOVA F: statistics F; p: descriptive level; CA: Anterior semicircular canal; CL: Lateral semicircular canal; CP: Posterior semicircular canal.

There was no statistically significant difference (p = 0.5213) between number of statoconia repositioning maneuvers necessary to make the positioning nystagmus disappear at Dix, Hallpike test, considering only the ANOVA factor “affected semicircular canal”, regardless of pathophysiological substrate (Table 3).

The interaction between factors that affected the semicircular canal and pathophysiological substrate showed statistically significant difference (p = 0.0652), as demonstrated in Table 3.

## DISCUSSION

BPPV is a labyrinthic condition of high prevalence, and even though it is labeled as benign, it may considerably affect quality of life of the patient and lead to social and/or professional disability. Thus, the application of efficient treatment is important to control symptoms. The therapeutic options are varied and we may use vestibular function suppressing drugs, surgical procedures and vestibular rehabilitation exercises^2^.

The first exercises of vestibular habituation, proposed by Cawthorne, increased the tolerance of the vestibular system to vertigo, but they did not treat the cause of dizziness^12^; drugs reduce the intensity of vertigo but they do not eliminate the crises, whereas surgical procedures such as neurectomy of singular and posterior semicircular canal occlusion are not exempt from complications. By better knowing the pathophysiological mechanics of BPPV, management becomes less aggressive and more effective. The theories of cupulolithiasis and ductolithiasis enabled the creation of statoconia repositioning maneuvers, which aim at cleaning the cupula and the duct of semicircular canals from statoconia debris. Maneuvers comprise sequences of head and body repositioning that moves the statoconia debris towards the utriculus.

The great popularity and acceptance of statoconia repositioning maneuvers are due to the simplicity of the technique, its non-invasive character, because it is well tolerated by the patients and normally free from complications^9^.

The age of patients with BPPV in the present study ranged from 17 to 83 years, a similar variation of that found in the study by Macias et al., in which age ranged from 20 to 93 years^8^.

The current study showed predominance of female gender in relation to male gender, in a proportion of 1.4: 1. Hormonal affections, more frequently found in women, could favor higher occurrence of BPPV in women, according to Guzmán et al.^13^.

The inclusion of patients in this study has followed some criteria of “BPPV certainty” described by Hughes, Proctor^14^, in which the patients should present vertigo and the characteristic positioning nystagmus, with latency, limited duration and fatigue at diagnostic maneuver, contributing to differentiating BPPV from other clinical presentations of central, cervical or vascular origin that may cause positional vertigo and/or positioning vertigo^15^.

The diagnosis was defined by observation of vertigo and nystagmus at Dix, Hallpike test. According to Herdman, Tusa^6^ this maneuver may be used for the diagnosis of BPPV of any semicircular canal, even though he described the Roll maneuver as the most sensitive for the diagnosis of lateral semicircular canal. Frenzel lenses were used during the performance of Dix, Hallpike maneuver to sensitize the observation of the nystagmus, because they are useful for excluding the effects of ocular fixation, they enlighten and expand the image of the eyes.

Out of 60 studied subjects, 26 patients (56.7%) had hypothesis of cupulolithiasis and 34 patients (43.3%) of ductolithiasis. In the same sample, 46.6% presented affection of the posterior semicircular canal, 30% of the anterior semicircular canal and 23.3% of the lateral semicircular canal. Honrubia et al.^7^ presented different results in relation to proportion of patients in each physiological substrate and affected semicircular canal, upon studying 269 patients with unilateral BPPV, out of which 263 (97.77%) had ductolithiasis as hypothesis and 4 patients (2.2%) had cupulolithiasis as hypothesis. Similarly, they found 250 patients (92.93%) with affection of posterior semicircular canal, 4 patients (1.49%) with affection of anterior semicircular canal, and 15 patients (5.58%) with affection of lateral semicircular canal.

The larger number of patients with cupulolithiasis relative to ductolithiasis, as well as the greater proportion of affection to anterior and lateral semicircular canals in our study in relation to the results published by Honrubia can be explained by the different inclusion criteria of the patients in these studies. The patients of our study were included consecutively for each combination between pathophysiological substrate and affected semicircular canal; in the Honrubia study, the patients were consecutive regardless of the affected semicircular canal and pathophysiological substrate.

The efficacy of statoconia repositioning maneuvers in treating BPPV has been studied by many different authors^2^^,^^7^^,^^15^^,^^16^. Hilton, Pinder^17^, performed a systematic review on the repositioning maneuver for posterior semicircular canal and concluded, based on controlled double blind studies, that there is evidence that the maneuver is efficient for the treatment of BPPV. However, there are few studies that compare the efficiency of maneuvers relative to different semicircular canals and pathophysiological substrates.

In our sample, it required on average 2.13 repositioning maneuvers (ranging from one to 8) to make the positioning nystagmus disappear at Dix, Hallpike test. Wolf et al.^18^ needed an average of 1.23 maneuvers, Macias et al.^8^ required on average 1.36 maneuvers, Gans, Harrington-Gans^19^ required on average 1.3 maneuvers and Macias et al.^20^ needed 1.38. According to Vrabec^9^, the number of repositioning maneuvers required to induce remission of BPPV is variable and multiple treatment approaches may be necessary in more than a third of the patients.

Out of the total of patients in the present study, 40% required only one maneuver to have remission of positioning nystagmus, 28.3% required two maneuvers, and 41.7% needed three or more maneuvers. However, Wolf et al.^18^ treated 107 patients with BPPV and detected that 74,7% required one maneuver, 15.9% required two, 2.8% three maneuvers, and 6.6% did not improve with up to three maneuvers. Gans, Harrington-Gans^19^ treated 376 patients and reported resolution in 79% of the patients with one maneuver, in 17% of the patients they required two maneuvers, in 3.5% of the patients three maneuvers were necessary, and in 0.05% of the patients, four maneuvers were required. Macias et al.^20^, upon treating 102 patients with BPPV, observed that 70.6% of the subjects required only one maneuver to have remission of nystagmus and positioning vertigo at Dix, Hallpike test, 20.6% required two maneuvers and 8.8% needed three maneuvers.

The higher number of patients with BPPV by cupulolithiasis in the sample of the present study may have caused increase on the average of maneuvers and the number of necessary maneuvers to have remission of positioning nystagmus relative to the studies previously reported. According to Fung, Hall^21^ cupulolithiasis would be one cause of failure of repositioning maneuvers.

Despite the increase in mean number of statoconia repositioning maneuvers presented by the current study, at the end of treatment all patients had remission of positioning nystagmus with up to 8 maneuvers in their clinical follow up. These results are in agreement with high therapeutic efficiency of maneuvers, obtained by Epley, which reached 100% efficiency^15^.

When we considered only ANOVA factor of pathophysiological substrate, regardless of the factor affected canal, it took fewer repositioning maneuvers to have remission of positioning nystagmus for cupulolithiasis relative to ductolithiasis, with statistically significant difference. In cupulolithiasis, the theory created by Schuknecht, statoconia fractions would be firmly adhered to the cupula of semicircular canals, thus more difficult to be removed by repositioning maneuvers in relation to ductolithiasis, in which the same particles would be freely floating in the endolymph of semicircular canals.

When we excluded the ANOVA factor of pathophysiological substrate and considered only the factor of affected semicircular canal, we noticed that there was no significant difference. Korres et al.^15^ did not find any difference between number of necessary maneuvers for treatment when they compared the anterior, lateral and posterior canals. However, Macias et al.^8^ observed that patients with BPPV of anterior, lateral or bilateral affection of posterior canal are more likely to require greater number of statoconia repositioning maneuvers relative to patients with affection of only one posterior semicircular canal.

The association of pathophysiological substrate and affected semicircular canal factors showed a tendency to having statistically significant difference in the number of required maneuvers to have remission of positioning nystagmus by means of therapeutic maneuvers. The difference in number of therapeutic maneuvers is present only in anterior and posterior semicircular canals, and it is not present in the lateral canal. In posterior and anterior canals, ductolithiasis required on average a smaller number of maneuvers than cupulolithiasis. The lateral canal, both for ductolithiasis and cupulolithiasis, required the same number of repositioning maneuvers. We do not find relevant scientific studies in the literature that have analyzed the association between pathophysiological substrate and affected semicircular canal and their influence in number of necessary maneuvers to reach remission of positioning nystagmus.

Some factors could have influenced the results of this study, both to increase and to reduce the number of therapeutic maneuvers required to reach remission of positioning nystagmus. Failure in treatment or greater number of repositioning maneuvers required for the success of treatment may be owed to many different factors, including cupulolithiasis, atelectasia of vestibular membranous labyrinth and inappropriate repositioning maneuvers. The degeneration of utriculus macula or detachment of otolithic membrane would cause failure in therapeutic maneuver^22^. Aranda-Moreno, Jáuregui-Renaud^23^ mentioned the fact that the association of BPPV and other labyrinthic affections may increase the number of required therapeutic maneuvers. Gross et al.^22^ described nine cases of patients with Meniere disease associated with BPPV that did not respond to therapeutic maneuvers. In such cases, repeated distensions of the membranous labyrinth caused by endolymphatic hypertension would cause a collapse of the labyrinth or would facilitate the adhesion of particles, which would prevent the removal from the semicircular canals.

Spontaneous remission of vertigo and positioning nystagmus is a factor to be considered, because it may have increased the index of therapeutic success in the present study. The index of spontaneous remission found in Lynn et al.^24^ was of 27%. According to Vrabec^9^, there may be spontaneous resolution of BPPV by absorption of statoconia, but the factors responsible for induction or prevention are very poorly known.

## CONCLUSIONS

• It took one to eight weekly repositioning maneuvers, on average two, to cause remission of positioning nystagmus in patients with BPPV.
• Cupulolithiasis required greater number of maneuvers than ductolithiasis to cause remission of positioning nystagmus. The affected semicircular canal did not influence the number of therapeutic maneuvers.
